# A Unique Case of Intracranial Bifocal Germinoma

**DOI:** 10.7759/cureus.62167

**Published:** 2024-06-11

**Authors:** Tarani Chetana Naga Sai, Ramakrishna Narayanan, Megha S Uppin, Ramanadha Reddy Kanala

**Affiliations:** 1 Radiology, Nizam's Institute of Medical Sciences, Hyderabad, IND; 2 Pathology, Nizam's Institute of Medical Sciences, Hyderabad, IND; 3 Neurological Surgery, Nizam's Institute of Medical Sciences, Hyderabad, IND

**Keywords:** bifocal, chemoradiation, panhypopituitarism, pineal, suprasellar, germinoma

## Abstract

Primary intracranial germ cell tumors are rare tumors that often occur in children and young adults. We report a case of a 17-year-old male, who presented with vomiting, headache, and blurring of vision of the left eye on the temporal aspect for two months. His biological assessment showed panhypopituitarism. Serum markers showed elevated beta human chorionic gonadotropin and lactate dehydrogenase. A solid cystic lesion was noted on imaging, involving the sella, parasellar region, and pineal region with calcifications within. Diagnosis of bifocal germinoma was confirmed by tumor biopsy. The treatment protocol for the patient involved four cycles of chemotherapy using etoposide and carboplatin, followed by a course of radiotherapy.

## Introduction

Primary germ cell tumors of the central nervous system are rare tumors that occur due to aberrant migration of primordial germ cells during embryogenesis. They look similar to the gonadal germinomas on histopathology.

Germ cell tumors are broadly classified into two types: seminomatous and non-seminomatous tumors. Seminomas (germinomas) are the most common germ cell tumors and non-seminomatous germ cell tumors include embryonal carcinoma, yolk sac tumor, choriocarcinoma, teratoma, and mixed germ cell tumors [1].

Germinomas account for 3-5% of pediatric intracranial tumors. More than 90% of these tumors occur in children less than 20 years of age. The most common location is the pineal gland. Gender ratios show marked male preponderance (22:1 in the pineal region) and mild female preponderance when involving the suprasellar region [2].

Bifocal germinomas are treated as primary germinomas and have a good prognosis (>90% remission) [3].

## Case presentation

A 17-year-old male patient with no prior comorbidities came with complaints of headache, vomiting, and reduced vision of the left eye for two months. The patient was apparently asymptomatic two months ago when he had vomiting, which was non-bilious and non-projectile, a headache, which was diffuse and dull aching, and reduced vision in the left eye.

On clinical examination, the patient was conscious, coherent, cooperative, afebrile, and normotensive. Visual field examination showed bitemporal hemianopia. Other systemic examinations were normal.

Further investigations showed an elevated serum osmolality. A pituitary gram showed low serum cortisol, low prolactin, low follicle-stimulating hormone (FSH), low luteinizing hormone (LH), low triiodothyronine (T3), low thyroxine (T4), low thyroid-stimulating hormone (TSH), low growth hormone, and low insulin-like growth factor-1 (IGF-1) (Table 1).

**Table 1:** Laboratory investigations of the patient. The biological profile revealed panhypopituitarism and elevated b-hCG and LDH levels. FSH: follicle-stimulating hormone; LH: luteinizing hormone; T3: triiodothyronine; T4: thyroxine; TSH: thyroid-stimulating hormone; IGF-1: insulin-like growth factor; b-hCG: beta human chorionic gonadotropin; LDH: lactate dehydrogenase; AFP: alpha-fetoprotein; mosm: milliosmoles; ug: micrograms; ng: nanograms; ml: milliliter; mIU: milli-international units; uIU: micro international units; U: units.

Parameters	Results	Reference value
Serum osmolality	312	275-295 mosm/L
Serum cortisol	1.5	Morning: 6.5-22 ug/dl
Serum prolactin	1.7	2.64-13.13 ng/ml
Serum FSH	0.2	1.2-19.2 mIU/ml
Serum LH	0.2	1.2-8.6 mIU/ml
Serum T3	0.6	0.8-2.1 ng/ml
T4	3.9	4.8-15.6 ug/ml
TSH	0.34	0.7-6.4 uIU/ml
Growth hormone	1	<7 ng/ml
IGF-1	77.3	193-731 ng/ml
b-hCG	83	≤5 mIU/ml
LDH	338	125-220 U/L
AFP	2.98	<7 ng/ml

Tumor markers assay revealed elevated beta human chorionic gonadotropin (b-hCG) and lactate dehydrogenase. Serum alpha-fetoprotein (AFP) levels were normal.

**Figure 1:**
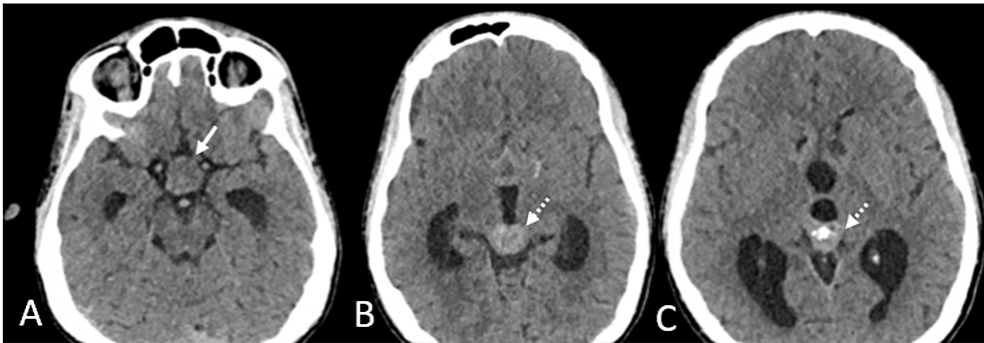
Unenhanced axial CT images of the brain at the level of the suprasellar cistern (A) and the level of the third ventricle (B, C) showed an isodense lesion in the suprasellar region (white arrow) and a hyperdense lesion (dashed arrow) with central “engulfed” calcification in the pineal gland region.

On radiological assessment, unenhanced computed tomography (CT) (Figure 1) revealed a well-defined isodense lesion in the sella extending into the parasellar and suprasellar regions and involving the hypothalamus. There was another well-defined hyperdense lesion with coarse central calcifications in the pineal gland obstructing the third ventricle and causing obstructive hydrocephalus of the ventricular system.

**Figure 2:**
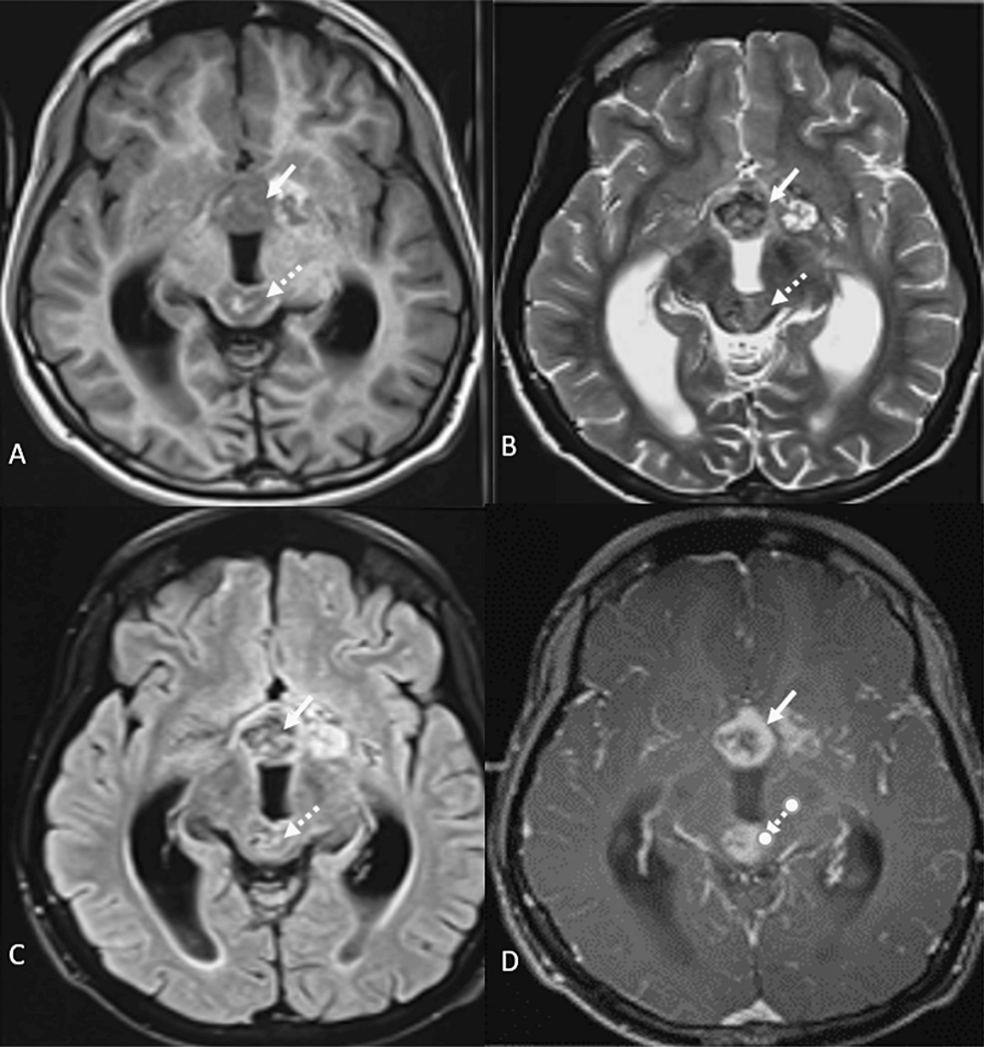
(A) MRI of the brain axial-T1 weighted image showed that the lesion involving the sella, suprasellar (bold arrow) and parasellar regions, and pineal gland (dotted arrow) was heterogeneously hypointense on T1-weighted images with few hyperintense areas within. (B and C) MRI of the brain axial T2-weighted and fluid-attenuated inversion recovery (FLAIR) images, respectively, showed a heterogeneously hypointense lesion involving the sella, suprasellar (bold arrow), and parasellar locations, and pineal gland (dotted arrow). (D) MRI of the brain axial post-contrast image showed that the suprasellar (bold arrow) and pineal gland (dotted arrow) lesions showed heterogenous enhancement on the post-contrast image with central non-enhancing areas.

**Figure 3:**
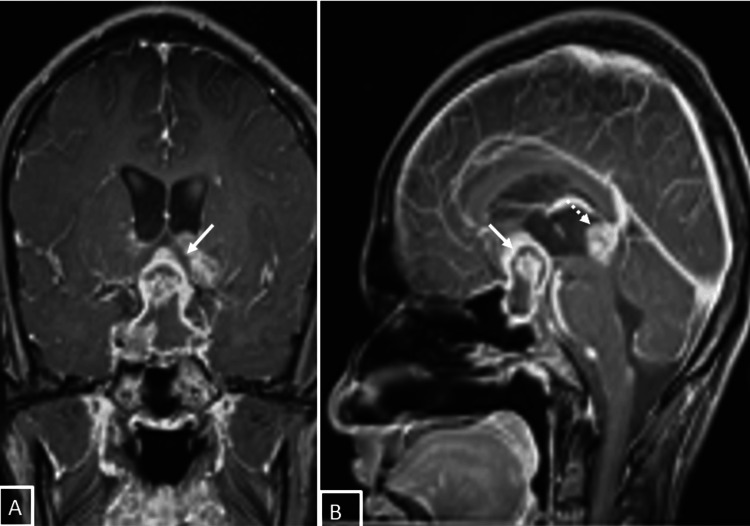
MRI of the brain (A) coronal and (B) sagittal post-contrast images showed a heterogeneously enhancing lesion involving the sella and suprasellar region (bold arrow) and another similar lesion in the pineal gland (dotted arrow).

The patient was then referred for magnetic resonance imaging (MRI). MRI (Figures 2, 3) showed that the lesion was a well-defined, solid cystic lesion, involving the suprasellar region extending into the sella, and the parasellar region and hypothalamus. Another lesion with similar signal characteristics was also noted in the pineal gland. Both lesions showed heterogenous enhancement on post-contrast imaging.

Based on the CT and MRI features, differentials of bifocal germinoma, primitive neuroectodermal tumors (PNET), and germinoma with metastasis were considered.

**Figure 4:**
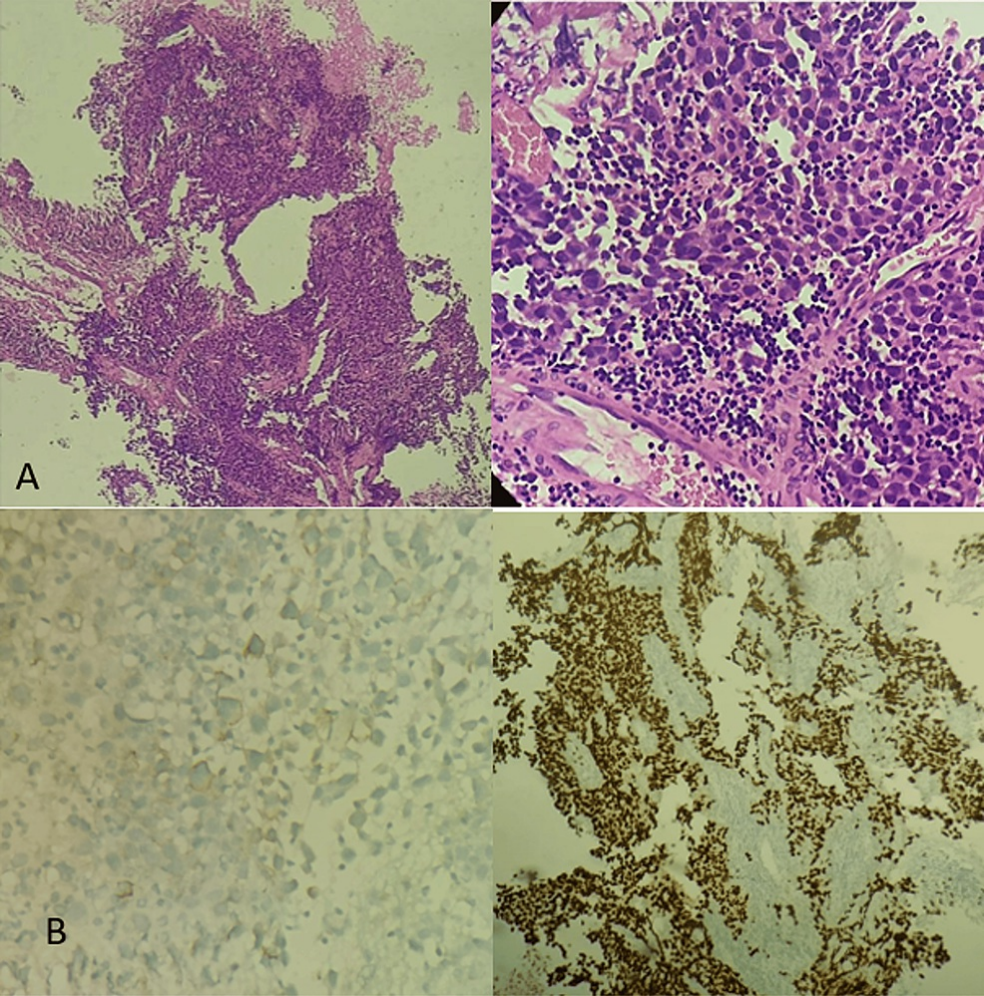
(A) The biopsy specimen with hematoxylin and eosin staining under 10x magnification revealed a lesion arranged in lobules separated by thin fibrous septae. The septae showed lymphocytic infiltrate. The lesional cells are polygonal and show vesicular nuclei with prominent nucleoli and a moderate amount of cytoplasm. Atypia is also seen and a diagnosis of germinoma is made. (B) Immunohistochemistry revealed CD117 and SALL4 positivity.

MRI of the whole spine was done to look for drop metastasis, which showed no drop metastasis. Endoscopic transventricular biopsy of the lesion (Figure 4) and external ventricular drainage (EVD) (Ommaya reservoir) placement was done.

**Figure 5:**
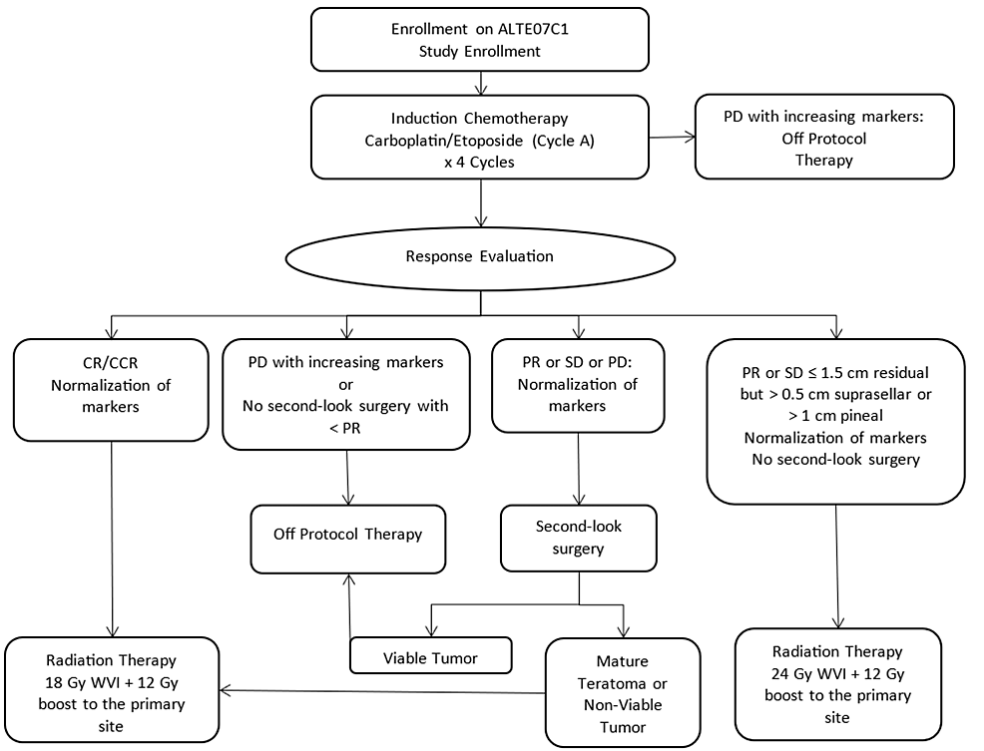
Flow chart showing the treatment plan. Based on the guidelines from Takami et al. [4]. CR: complete resolution; CCR: clinical complete response; WVI: whole ventricular irradiation; PD: progressive disease; PR: partial resolution; SD: stable disease.

The plan of treatment was a combination of chemotherapy involving four cycles of carboplatin and etoposide and radiotherapy (Figure 5).

## Discussion

Most germ cell tumors occur in patients under 20 years of age, mostly in adolescents, and are common in boys. Bifocal tumors in the pineal and suprasellar regions are not considered metastatic [1].

Clinical signs depend on the size and location of the tumor. Acutely, it can present with hydrocephalus with signs of raised intracranial pressure. In the suprasellar location, it can present with optic symptoms like blurring of vision and visual field alteration and neuroendocrine symptoms like growth retardation, delayed puberty, hypothyroidism, diabetes insipidus, and panhypopituitarism [1].

Specific tumor markers like elevated alpha-fetoprotein indicate the presence of a yolk sac tumor, and elevated b-hCG indicates the presence of a germinoma or choriocarcinoma.

On imaging, germ cell tumors often develop in the midline (pineal and suprasellar regions in unifocal or bifocal form), or can involve the thalamus. In the basal ganglia, they can be cystic/hemorrhagic masses. Germinomas usually have calcifications and sometimes present with hemorrhage. On MRI, germinomas are isointense to hyperintense on T2-weighted sequences with areas of cystic changes and enhancing of solid components on post-contrast images. They also show low apparent diffusion coefficient values on diffusion-weighted images [5].

On histopathology, germinomas consist of larger cells with epitheloid cells, which are abundantly positive for periodic acid-Schiff (PAS), highly positive for placental alkaline phosphatase, express CD117 and OCT4 positivity, and may show positive b-hCG [6].

A complete assessment includes a high-resolution ultrasound of both testes, PET-CT to rule out metastasis, a neuroophthalmological assessment, and a fertility preservation consultation before starting chemotherapy.

The treatment for germinoma includes endoscopic ventriculostomy or ventriculoperitoneal or an external shunt is performed for hydrocephalus. As germinomas are very sensitive to chemotherapy and radiotherapy, excisional surgery is not recommended. The prognosis is excellent in cases with no metastasis with a cure rate of about 90%. In metastatic forms, craniospinal radiotherapy without chemotherapy is recommended [7]. Surgical resection in germinoma is usually reserved when residual mass remains after treatment, which suggests a mixed tumor, for example, a combination of teratoma and germinoma [8].

Follow-up needs to be done with an MRI of the brain every four to six months for the first three years and then yearly thereafter. A spinal MRI is indicated if spinal lesions have been identified previously or the patient has symptoms related to the spine. When relapse occurs, tumors tend to occur outside the radiation field via subependymal or CSF spread. In cases of relapse, thiotepa-based or melphalan-based high-dose chemotherapy regimens, followed by autologous stem cell rescue, can be used [8]. In cases with metastasis, craniospinal radiotherapy without chemotherapy is recommended [9]. New therapeutic strategies are aimed at methods to reduce the dose of radiation and chemotherapy [10].

## Conclusions

Intracranial germinomas usually occur in young adults and show a strong male preponderance. However, the occurrence of bifocality in these tumors is exceedingly rare. Therefore, when encountering masses in the suprasellar and pineal regions, radiologists must consider the possibility of bifocal germinoma among the differentials. Early detection and treatment are pivotal in reducing morbidity and mortality. Through a comprehensive approach combining chemotherapy and radiotherapy, patients can expect a favorable prognosis, with cure rates exceeding 90%.
